# Endothelium-Derived Hyperpolarizing Factor and Vascular Function

**DOI:** 10.4061/2011/156146

**Published:** 2011-08-15

**Authors:** Muhiddin A. Ozkor, Arshed A. Quyyumi

**Affiliations:** ^1^The Heart Hospital, University College London, London WIG 8PH, UK; ^2^Division of Cardiology, Department of Medicine, Emory University, 1364 Clifton Road NE, Suite 403C, Atlanta, GA 30322, USA

## Abstract

Endothelial function refers to a multitude of physiological processes that maintain healthy homeostasis of the vascular wall. Exposure of the endothelium to cardiac risk factors results in endothelial dysfunction and is associated with an alteration in the balance of vasoactive substances produced by endothelial cells. These include a reduction in nitric oxide (NO), an increase in generation of potential vasoconstrictor substances and a potential compensatory increase in other mediators of vasodilation. The latter has been surmised from data demonstrating persistent endothelium-dependent vasodilatation despite complete inhibition of NO and prostaglandins. This remaining non-NO, non-prostaglandin mediated endothelium-dependent vasodilator response has been attributed to endothelium-derived hyperpolarizing factor/s (EDHF). Endothelial hyperpolarization is likely due to several factors that appear to be site and species specific. Experimental studies suggest that the contribution of the EDHFs increase as the vessel size decreases, with a predominance of EDHF activity in the resistance vessels, and a compensatory up-regulation of hyperpolarization in states characterized by reduced NO availability. Since endothelial dysfunction is a precursor for atherosclerosis development and its magnitude is a reflection of future risk, then the mechanisms underlying endothelial dysfunction need to be fully understood, so that adequate therapeutic interventions can be designed.

## 1. Introduction

Endothelial function refers to a multitude of physiological processes of the vascular endothelium that maintain healthy homeostasis of the vascular wall and may be used as a “barometer” of the injury/repair inflicted by multiple environmental and genetic factors [1–3]. Vascular endothelial dysfunction is associated with a reduction in nitric oxide (NO) bioavailability, an increase in generation of potential vasoconstrictor substances such as superoxide anions and endothelin–1, and a potential compensatory increase in other mediators of vasodilation. This remaining non-NO, nonprostaglandin-mediated endothelium-dependent vasodilation has been partly attributed to endothelium-derived hyperpolarizing factor/s (EDHF). Endothelial hyperpolarization is likely due to several factors that are site- and species-specific, ultimately causing vascular smooth muscle hyperpolarization and relaxation. Experimental studies suggest that the contribution of EDHFs increase as the vessel size decreases, with predominant EDHF activity in the resistance vessels and a compensatory upregulation of EDHFs in states characterized by reduced NO availability [4–12]. Whereas prostacyclin and NO bioavailability have been extensively investigated in the human circulation *in vivo*, little is known about endothelial hyperpolarization. 

## 2. Endothelium-Derived Hyperpolarizing Factor (EDHF)

**Figure 1:**
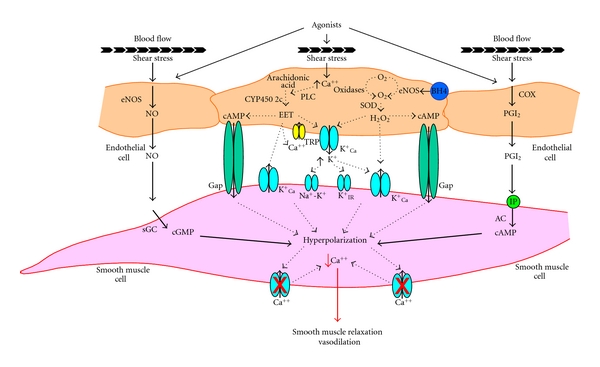
Mechanisms for endothelial cell mediated relaxation. Agonist (bradykinin/acetylcholine/substance P) or shear stress increases the activity of endothelial NO synthase (eNOS) and cyclooxygenase (COX), providing nitric oxide x(NO) and prostacyclin(PGI_2_)-mediated dilation. There are multiple potential EDHF pathways. Increases in intracellular calcium activates phospholipase A2 (PLC) to produce arachidonic acid. Its metabolism by cytochrome P450 2C (CYP4502c) generates eicosatrienoic acids (EETs) that can stimulate calcium dependent potassium (K_Ca_^+^) channels in endothelial and smooth muscle cells. EETs may also directly activate gap junctions (Gap). EETs may also act in an autocrine manner on endothelial cells by activating transient receptor potential (TRP) V4 channels, which promote Calcium (Ca^++^) influx further increasing the calcium concentration and activating K_Ca_^+^ channels to cause hyperpolarization and release of K^+^ ions into the subendothelial space. The increase in potassium in the interstitium may activate K_Ca_^+^ channels, inwardly rectifying potassium channels (K_IR_^+^), or the Na^+^-K^+^ pump on smooth muscle cells and cause hyperpolarization. Smooth muscle hyperpolarization in turn results in relaxation by closing voltage-gated channels leading to a fall in Ca^++^ concentration and subsequent vasodilation. The action of eNOS (with cofactor tetrahydrobiopterin [BH4]) and oxidases on oxygen (O_2_) produces the reactive oxygen species superoxide (^.^O_2_−). Hydrogen peroxide (H_2_O_2_) generated by dismutation of superoxide anions by superoxide dismutase (SOD) can also cause hyperpolarization by activating endothelial and smooth muscle K_Ca_^+^  channels or by gap junctions. Adenylyl cyclase: AC; cyclic Adenosine monophosphate: cAMP; cyclic guanosine monophosphate: cGMP; soluble guanylyl cyclase: sGC; prostacyclin receptor, IP.

Potential EDHFs differ by species and vascular bed, but act by increasing potassium (K^+^) conductance resulting in the subsequent propagation of depolarization of vascular smooth muscle cells and relaxation [13–15] (Figure 1). Acetylcholine causes hyperpolarization of vascular smooth muscle in arteries with an intact endothelium but not in its absence [16–19]. This hyperpolarization is mimicked by certain K^+^ channel agonists and is unaffected by inhibitors of nitric oxide synthase or cyclooxygenase and has been attributed to release of EDHF/s. EDHFs appear to open smooth muscle cell K^+^ channels, allowing K^+^ efflux along its chemical gradient resulting in membrane hyperpolarization. Thus, EDHF activity may be defined functionally as agonist-induced, endothelium-dependent relaxation that is not blocked by inhibitors of NO synthase or cyclooxygenase but can be inhibited, at least in part, by K^+^ channel blockers.

### 2.1. Potassium (K_Ca_^+^) Channel Activation (Figure 1) 

#### 2.1.1. Calcium-Activated Potassium (K_Ca_^+^) Channels

Agonists such as bradykinin stimulate endothelial G protein-coupled receptors provoking an increase in intracellular calcium [Ca_2+_]*_i_* in the endothelial cell [20, 21]. Endothelium-dependent hyperpolarization of smooth muscle cells results from the opening of K^+^ channels in the smooth muscle plasmalemma and is abolished by K^+^ concentrations higher than 25 mM [22]. Agonists that produce hyperpolarization also stimulate efflux of K^+^ [17, 23]. However, there are several smooth muscle K^+^ channels; endothelium-dependent hyperpolarization is not prevented by glibenclamide, an inhibitor of ATP-dependent K^+^ channels (K_ATP_^+^), or inhibitors of inwardly rectifying potassium channels (K_IR_^+^) [24]. However, both barium chloride (<100 *μ*mol/L) and ouabain (2.7 nmol/min), inhibitors of K_IR_^+^ channels, reduced resting flow in healthy subjects, an effect that was lower in obesity, even though these are not endothelium-dependent responses [25].

The hallmark of the EDHF-mediated responses is its abolition by the combination of apamin (a specific inhibitor of K_Ca_^+^ channels of small conductance (SK_Ca_^+^ channels)) plus charybdotoxin (a nonselective inhibitor of large-conductance (BK_Ca_^+^) and intermediate-conductance (IK_Ca_^+^) channels), and of some voltage-dependent K^+^(K_V_^+^) channels [26–28]. This toxin combination targets K_Ca_^+^ channels on endothelial cells rather than K^+^ channels located on smooth muscle cells. Increasing intracellular free calcium in endothelial cells opens K_Ca_^+^ channels allowing efflux and accumulation of K^+^ into the myoendothelial space. This triggers several processes that explain the EDHF phenomena; including (1) synthesis of cytochrome that P450 (CYP450) metabolites, a family of epoxides, (2) transmission of endothelial cell hyperpolarization to the vascular smooth muscle via gap junctions, and (3) K^+^ released from the endothelial cells via K_Ca_^+^ channels induces smooth muscle hyperpolarization by activating K_Ca_^+^ channels and/or Na^+^-K^+^-ATPase on vascular smooth muscle cells. 

Study of gastroepiploic conductance arteries and microvessels revealed that the contribution of EDHF to endothelium-dependent relaxations is significantly larger in human microvessels than in large arteries, that EDHF-mediated relaxations are mediated via activation of K^+^ channels, and that risk factors, particularly hypercholesterolemia and aging, were associated with impaired EDHF-mediated dilation [4]. Convincing evidence has been presented to suggest that a CYP450-dependent EDHF plays a significant role in the regulation of coronary arteriolar tone by K_Ca_^+^ channel activation and smooth muscle hyperpolarization [8, 29]. Thus, L-NG-nitro arginine/indomethacin-insensitive component of acetylcholine-mediated relaxation was sensitive to 25 mmol/L K^+^, but not to glibenclamide, a K_ATP_ channel inhibitor. Importantly, relaxation in human subcutaneous resistance arteries was abolished by a combination of charybdotoxin and apamin indicating the crucial role for endothelial K_Ca_^+^ channel activation [7].

Human studies have used tetraethylammonium chloride (TEA) to investigate the role of large K_Ca_^+^ channel activation on forearm blood flow and on the vascular effects of bradykinin. TEA selectively blocks K_Ca_^+^ channels in arterial smooth muscle cells at concentrations of  >1 mmol/L [29]. At these doses, TEA decreased resting forearm blood flow by 23% and radial artery diameter by 5%, and it also inhibits bradykinin- and substance P-induced, but not acetylcholine-mediated vasodilation after inhibition of NO and prostaglandins in the forearm microcirculation [30–32]. Moreover, resting radial arterial blood flow and diameter were reduced only with combined blockade of NO synthesis and K_Ca_^+^ channels but not with either blockade individually, suggesting an important interaction between these two vasodilator systems [31]. These observations demonstrate the contribution of both NO and K_Ca_^+^ channel activation to resting conductance artery and microvascular tone in the healthy human forearm circulation.

### 2.2. Epoxyeicosatrienoic Acids (EETS) (Figure 1)

Epoxyeicosatrienoic acids (EETs) are arachidonic acid derived products of cytochrome P450 (CYP450) epoxygenases [33]. CYP450 enzymes are membrane-bound, heme-containing terminal oxidases in a multienzyme system. The arachidonic acid metabolizing CYP450 enzymes with prominent roles in vascular regulation are the epoxygenases of the CYP 2 gene family (e.g., CYP 2B, 2C8, 2C9, 2C10, and 2J2 in humans) that generate a series of region-specific and stereo-specific epoxides (5,6-, 8,9-, 11,12-, and 14,15-EETs), and the arachidonic acid v-hydroxylases belonging to the CYP 4A family which form hydroxyeicosatetraenoic acids (HETEs) which can demonstrate organ specific opposed actions [34]. 

Evidence in favor of EDHF being a short-lived metabolite of the cytochrome P450 epoxygenase pathway has been obtained using bovine [35, 36], porcine [36, 37], canine [38, 39], murine [40, 41], and human coronary arteries [8, 9, 29, 42–45]. EDHF-mediated responses are blocked by nonspecific CYP450 inhibitors such as miconazole, 17-octadecynoic acid, and more selective epoxygenase inhibitors [6-(2-proparglyloxyphenyl) hexanoic acid and *N*-methylsulphonyl-6-(2-propargyloxyphenyl) hexanamide] [46–48]. RT-PCR, Western blotting, and immunofluorescence techniques have demonstrated that native coronary endothelial cells express CYP epoxygenases, including CYP 2C8, CYP 2C9, and CYP 2J2 [49–52]. EET-induced activation of K_Ca_^+^ channels appears to be mediated by a cascade of intracellular events involving the ADP ribosylation of cellular proteins including anti-G(S)alpha antibody [53, 54] ultimately hyperpolarizing smooth muscle cells by increasing the open-state probability of K_Ca_^+^  channels [35, 41, 50, 55]. In porcine coronary arteries, a CYP450-derived epoxide, namely 11,12-epoxyeicosatrienoic acid (11,12-EET) has been shown to possess EDHF properties because (1) both 11,12-EET and bradykinin elicit hyperpolarization; (2) endothelial cells, but not smooth muscle cells, expressed mRNA and protein for the epoxygenase enzyme of the CYP2C family and released 11,12-EET; (3) induction of CYP2C8 or CYP2C34 increased epoxygenase expression, which was associated with increased release of 11, 12-EET, and enhanced relaxation and hyperpolarization in response to bradykinin; (4) an antisense oligonucleotide directed at the endothelial epoxygenase reduced both CYP2C mRNA and protein expression and the capacity to generate 11,12-EET, concomitantly with a reduction in the vasorelaxant and hyperpolarizing response to bradykinin [50]. The finding that sulfaphenazole, a selective inhibitor of CYP 2C9 [56, 57], inhibits EDHF-mediated responses [50] and potentiates non-NO-mediated relaxation in the porcine coronary artery [33] suggests that the CYP isoform required for the generation of EDHF is a porcine equivalent of CYP 2C9 [58]. Further, there is evidence in some species that CYP450-derived epoxides stimulate Na^+^/K^+^ ATPase [59]. These data strongly suggest that the activation of a cytochrome P450 epoxygenase is a prerequisite for the generation of EDHF-mediated relaxation in certain species. 

Other intracellular second messenger roles of EETs may be equally as important in the control of vascular homeostasis. EETs (in particular 11,12- and 14,15-EET) activate several intracellular protein kinases including tyrosine kinases, the p38 MAP kinase, and extracellular-regulated protein kinases 1 and 2 (Erk1/2) and increase the proliferation of various cell types, including vascular smooth muscle cells and endothelial cells [33, 60, 61].

In isolated human coronary arterioles, CYP450-dependent hyperpolarization plays a significant role in the regulation of smooth muscle tone via activation of K_Ca_^+^  channels [8, 29]. Human coronary arteriolar endothelium-dependent hyperpolarization in response to arachidonic acid is much more dependent on metabolism by CYP450 than by cyclooxygenase or lipoxygenase or activation of NO synthase [8, 62]. The predominant EET synthesized by arteries is 11,12-EET, and its specific inhibition by chemically distinct CYP450 inhibitors impairs EDHF relaxation. 11,12-EET activates large-conductance K_Ca_^+^ channel current and hyperpolarizes arterial smooth muscle. Large-conductance K_Ca_^+^ channels and CYP450-2C mRNA and proteins are less abundant in arteries than saphenous veins explaining the lack of EDHF activity of veins. Large-conductance K_Ca_^+^ channels are primarily present in vascular smooth muscle, whereas the CYP450-2C enzyme is present in both the endothelium and smooth muscle cells. Thus, in human internal mammary arteries, EDHF appears to be 11,12-EET, produced by an EDHF synthase CYP450-2C and accounts for 40% of net endothelial relaxation by stimulating large-conductance K_Ca_^+^ channels [6].

The role of EETs as potential EDHFs can be studied using azoles such as miconazole that selectively inhibit epoxidation (EET generation) of arachidonic acid and have been demonstrated to be partly responsible for endothelium-dependent vasodilation in the human microcirculation [9, 31, 63]. *In vivo* studies have demonstrated CYP450 inhibition does not alter conductance vessel diameter or resting blood flow [31, 63, 64], but after inhibition of NO and prostacyclin, inhibition of EET synthesis further decreases radial arterial blood flow and diameter [31]. Thus, although it appears that under resting conditions in the healthy human forearm, conductance and resistance vessel tone is not modulated by tonic activity of CYP450-derived epoxides, their role becomes evident after inhibition of NO and prostacyclin synthesis, illustrating the potential compensatory role of EETs on maintenance of basal tone when NO availability is diminished. 

In recent studies, we have addressed previous controversies regarding the contribution of EDHF to resting vasodilator tone. In the largest cohort studied to date, an important contribution of EDHF, via activation of K_Ca_^+^ channels, to resting microvascular dilator tone in the human forearm* in vivo *has been demonstrated [65]. For the first time, we have also demonstrated that cytochrome P450-derived epoxyeicosatrienoic acids also contribute to resting vasodilator tone in the healthy microcirculation with the use of fluconazole to block their action. We found a relatively greater contribution of NO compared to EDHF in the maintenance of resting vasodilator tone in the healthy human forearm microvasculature. The contribution of these two endogenous vasodilators to resting tone differed in subjects exposed to risk factors for atherosclerosis, in whom the vasculature is characterized by decreased NO bioavailability. We found preserved contribution of EDHF that appears to contribute equally as much as NO to resting vasodilator tone in subjects with risk factors. In the presence of NO blockade with L-NMMA, epoxyeicosatrienoic acid-mediated microvascular vasodilation also appeared to be upregulated in healthy subjects [65]. This indicates potential cross-talk between the NO and EET pathways, such that EET activity is upregulated in the setting of NO deficiency which may be demonstrated by blocking NO synthesis in the healthy circulation. Interestingly, in the risk factor group, there was a similar contribution of EETs to resting tone in the presence and absence of NO blockade. Such compensation may be crucial in maintaining normal resting blood flow in nonhypertensive patients with risk factors.

Finally, by conducting experiments using single and combined blockade, we also demonstrated that K_Ca_^+^ channel activation contributes to microvascular dilator tone even after inhibition of epoxyeicosatrienoic acids. This indicates that sources other than epoxyeicosatrienoic acids contribute to hyperpolarization of the resting human forearm microcirculation. Potential candidates which have been investigated in experimental studies, include hydrogen peroxide, potassium ions, and gap junctions. These alternate pathways warrant further investigation *in vivo* in humans.

### 2.3. Hydrogen Peroxide

Hydrogen peroxide also activates calcium-dependent potassium channels and remains a contender as an EDHF [66] (Figure 1). Reactive oxygen species can increase K^+^ channel activity and hyperpolarize smooth muscle [67, 68], and hydrogen peroxide may function as an EDHF [69]. Rubanyi and Vanhoutte reported that superoxide attenuates endothelium-dependent relaxations and that hydrogen peroxide causes endothelium-dependent and -independent relaxations [70]. Matoba et al. utilized catalase, an endogenous peroxidase to show inhibition of EDHF-mediated, endothelium-dependent relaxations and hyperpolarizations, resistant to indomethacin or N(omega)-nitro-l-arginine [71]. These findings have been confirmed in piglet pial arteries, canine subepicardial coronary arteries and arterioles, and during flow-induced vasodilation in human mesenteric arteries and coronary microvessels [66, 72–74].

Vascular endothelial cells have a capacity to produce superoxide and hydrogen peroxide from several intracellular sources, including endothelial NO synthase, cyclooxygenases, lipoxygenases, cytochrome P-450 epoxygenases, NAD(P)H oxidases, and xanthine oxidase [71, 75–78]. Flow-mediated dilation involves generation of superoxide originating from mitochondria and shear stress elicits luminal release of mitochondrial ubisemiquinone, a source for generating superoxide and hydrogen peroxide via metabolic processes occurring between complex I and complex III of the electron transport chain [79]. Although reactive oxygen species appear to fit the profile of EDHF, their physiologic role remains a subject of debate particularly in some human arteries because in human radial and internal mammary arteries, neither catalase nor superoxide dismutase inhibited relaxations to carbachol [80, 81]. 

Hydrogen peroxide also mediates hyperpolarization via activation of endothelial K^+^  channels, however, many species variations exist in the type of K^+^  channels that are activated [67, 68, 72, 73, 82–84]. Importantly, in human coronary microvessels, K^+^_Ca_  channels sensitive to charybdotoxin plus apamin appear to mediate hyperpolarization [73, 85]. In mouse mesenteric arteries, the inhibitory effect of catalase was unmasked by the inhibition of NO production, and vice versa, suggesting that NO and EDHF (hydrogen peroxide) compensate for each other [71, 86–89]. In canine subepicardial coronary arteries and arterioles, the response to acetylcholine and hypoxia was inhibited by L-NMMA primarily in subepicardial coronary arteries, whereas combined infusion of L-NMMA plus catalase or tetraethylammonium attenuated the vasodilator responses of coronary arteries of both sizes, demonstrating the predominance of hydrogen peroxide-mediated hyperpolarization in microvessels [74].

### 2.4. Gap Junctions

The EDHF phenomenon may be further explained by the transmission of endothelial cell hyperpolarization to the vascular smooth muscle via gap junctions [90–92]. These are myoendothelial and heterocellular. They couple endothelial cells to other endothelial cells and to smooth muscle cells, providing a low-resistance electrical pathway between the cell layers. Gap junctions are formed by the docking of two connexons present in adjacent cells that creates an aqueous pore permitting the transfer of ions and electrical continuity that establishes a uniform membrane potential across cells [93, 94]. Their number increases with diminution in the size of the artery [95], paralleling the importance of EDHF to vessel size with a greater influence in the resistance than in the conductance vessels [96].

Investigation of gap junctions as other potential EDHF mechanisms has been limited in man due to the lack of specific pharmacological agents. Rotigaptide, that enhances communication via the connexin 43 gap junction subunit, had no effect on basal vascular tone endothelium-dependent (bradykinin), -independent vasodilation, or t-PA release in the forearm arterial circulation of healthy men [97]. 

### 2.5. Potassium (K^+^)

A moderate increase in the myoendothelial K^+^ concentration can in some species [98] induce hyperpolarization of vascular smooth muscle cells by activating the inwardly rectifying K^+^  channels and the Na^+^/ K^+^ ATPase [24, 99, 100]. However, it is unlikely that K^+^ *per se* is EDHF.

## 3. Interactions between EDHF, Nitric Oxide, and Prostacyclin

The three main mediators of endothelial vasodilator function, NO, prostacyclin, and EDHF appear not to be mutually exclusive and act synergistically in a complex manner to maintain the health of the vasculature (Figure 1). In conduit arteries, NO is the predominant endothelium-derived vasodilator but has relatively less prominent contribution in the resistance vessels of the microcirculation where EDHF appears to predominate [96]. NO may tonically inhibit EDHF responses as some studies could only demonstrate EDHF responses once NO production had been inhibited [44]. 

## 4. EDHF and Disease

Experimental evidence indicates that a shift away from NO-mediated endothelium-dependent relaxation toward EDHF-dependent relaxation occurs in disease states [101, 102]. Alteration of EDHF-mediated responses has been reported with aging, hypertension, atherosclerosis, hypercholesterolemia, heart failure, angioplasty, eclampsia, diabetes, and sepsis. Depending on the vascular bed, this may either contribute to endothelial dysfunction or compensate for the loss of NO bioavailability [103–105]. In the human forearm of hypertensive subjects, Taddei and others demonstrated that endothelium-dependent vasodilation is maintained despite decreased NO bioavailability because of the compensatory increased activity of EDHF [25, 104, 105]. Hypercholesterolemia is generally associated with preserved EDHF responses where its enhanced activity may compensate for the decrease in NO-mediated relaxation [106–108]. Endothelium-dependent hyperpolarization appears to be inhibited in isolated gastroepiploic arteries from atherosclerotic patients, an effect that may be secondary to the duration of hypercholesterolemic injury [4]. In contrast, EDHF-mediated responses are depressed in some models of type I and type II diabetes with the exception of murine models [109]. 

In the healthy forearm microcirculation, we demonstrated that bradykinin-stimulated vasodilation is in part mediated by activation of K_Ca_^+^ channels and that the magnitude of contribution of NO was less than the contribution of K_Ca_^+^ channel activation [65]. Importantly, we found no contribution of K_Ca_^+^ channel activation to acetylcholine-stimulated vasodilation in healthy subjects, either in the presence or absence of NO-blockade. Thus, acetylcholine predominantly stimulates the release of NO and no EDHF, whereas bradykinin stimulates release of both EDHF and NO. We also demonstrated a similar contribution of NO and EDHF to bradykinin-mediated vasodilation in both groups. In contrast to effects of bradykinin, forearm blood flow response to acetylcholine was diminished in hypercholesterolemic subjects when compared to healthy subjects. Moreover, in hypercholesterolemia, we observed a significant contribution of K_Ca_^+^ channel activation and a lower NO release with acetylcholine that was distinctly different compared to the healthy circulation. Thus, while in health NO is the predominant contributor, in hypercholesterolemia, both NO and K_Ca_^+^ channel activation contribute equally to acetylcholine-mediated microcirculatory vasodilation [65]. 

Evidence suggests that CYP expression [110–112] and EET generation are increased in hypertension [113, 114], during salt loading [115], and in hypercholesterolemia [116]. Members of the CYP 2C family are inhibited by NO, a phenomenon that may explain why EDHF-mediated responses are barely detectable in the absence of the combined inhibition of NO synthases and cyclooxygenase in normal vessels. Thus the EET/EDHF pathway may be of less importance in healthy vessels and of greater significance in disease states where NO activity is impaired [58]. A similar phenomenon has been described for bradykinin-induced changes in forearm blood flow in essential hypertensive patients [25] and in arterioles removed from patients with coronary artery disease, where vasodilatation is mediated entirely by a mechanism sensitive to both CYP and K_Ca_^+^ channel inhibitors [9]. Such findings indicate that in the absence of NO, vascular tone can be regulated by an EDHF-like mechanism. Thus, whether EDHF plays a causal or compensatory role in the endothelial dysfunction in the human circulation remains to be elucidated.

## 5. Summary

Absence of consensus regarding the precise identity of EDHFs and a consequent lack of specific inhibitors has long hampered clinical translation of this phenomenon (Table 1). Recently, with improved understanding of the major signaling mechanisms underlying vascular hyperpolarization, the role of EDHF in the human circulation *in vivo* has begun to be dissected, but experimental pitfalls remain. These include the often nonspecific nature of the antagonists used, the concentrations and duration of action of these blockers are variable, and complete blockade cannot be achieved *in vivo, *even with high doses, because of the competitive nature of the antagonism. Nevertheless, an impressive body of knowledge has already emerged regarding the role of EDHF in the human circulation (Table 2).

Apart from its contribution to normal vascular physiology, the accentuated role of EDHF in diseased states is worthy of further investigation because CYP-450 expression and EET generation are increased in hypertension, during salt loading and in hypercholesterolemia. Vasodilation in essential hypertension [25], and in atherosclerotic coronary arterioles, is largely secondary to CYP and K_Ca_^+^ channel stimulation. There are also potential implications regarding disease susceptibility, with some polymorphisms within CYP epoxygenases being associated with an enhanced risk of developing coronary artery disease and hypertension [14]. What may ultimately be of even greater interest is development of specific agents targeting EDHF synthesis, understanding of other biological effects of EETs such as angiogenesis and modulation of cell growth, and their potential role in human disease [14].

## 6. Implications

Conventionally, endothelial dysfunction is characterized as a deficiency of NO activity, often secondary to exposure to cardiovascular risk factors. This leads to abnormalities in vasodilation and hence blood flow delivery. Because of the known protective role of NO on the vessel wall that impedes thrombosis and atherosclerosis, several strategies have been applied to improve NO availability/activity. Although replacing NO pharmacologically with NO donors is beneficial for symptomatic relief from coronary vasodilation, there appears to be no antiatherosclerotic effect of NO donors. Improving endothelial NO bioavailability with statins and angiotensin antagonists has nevertheless proven to be cardioprotective. What remains unknown is (a) whether enhancing EDHF in conditions with impaired NO activity would also be of therapeutic value, (b) whether agents that improve endothelial dysfunction (acetylcholine responses) such as statins and angiotensin antagonists, also enhance EDHF bioactivity, and (c) whether tissue plasminogen activator release is EDHF-dependent in health and disease. Indeed hypertension is associated with elevated epoxide hydrolase expression [128, 129], angiotensin II increases the expression of the epoxide hydrolase [130], and epoxide hydrolase inhibitors are effective in reversing the hypertensive effects of angiotensin II [128]. Thus, epoxide hydrolase inhibitors that increase epoxide levels and hence aid hyperpolarization need to be further investigated in subjects with endothelial dysfunction. 

Experimental studies indicate that cytochrome P450 expression and EET generation are increased in hypertension [104, 113], in hypercholesterolemia [116], and in atherosclerotic coronary arterioles [50, 64, 75]. Moreover, polymorphisms in the cytochrome P450 epoxygenase genes are associated with increased risk of coronary artery disease and hypertension [131, 132]. Thus, understanding the pathophysiology of endothelial dysfunction beyond NO, and in particular with respect to EDHF in these disease states, could be crucial in understanding both the pathophysiology of atherosclerosis and developing novel therapies.

## Figures and Tables

**Table 1:** Pharmacological inhibitors of EDHF. Pharmacological agents used as potential inhibitors of EDHF and their targets and limitations (modified from Torondel et al. [117]).

Pharmacological inhibitors	Targets	Comments
Apamin	SK_Ca_^+^	Highly specific
Charybdotoxin	IK_Ca_^+^-BK_Ca_^+^	Can inhibit some Kv channels
Iberiotoxin	BK_Ca_^+^	Highly specific
Tetraethylammonium	SK_Ca_^+^-IK_Ca_^+^-BK_Ca_^+^	Inhibit other K^+^ channels at >10^−2^) m
Tetraethybutylammonium	SK_Ca_^+^-IK_Ca_^+^-BK_Ca_^+^	Inhibit other K^+^ channels at >10^−2^ m
BaCl_2_	K_IR_^+^	—
Ouabain	Na^+^/K^+^ ATPase	Can affect gap junction activity at >10^−4^ m
KCL	K^+^ currents	Dilates at >10^−2^ m through K_IR_^+^ and Na^+^/K^+^ ATPase activation
18 *α*-glycyrrhetic acid	Gap junctions	Possesses nonjunctional effects on membrane currents
Connexin mimetic peptides	Gap junctions	Highly specific
Catalase	Hydrogen peroxide	—
17-octadecenoic acid	CYP	Inhibits the synthesis of the vasoconstrictor 20-HETE
Clotrimazole	CYP	Can inhibit K^+^ channels
Miconazole	CYP	Can inhibit K^+^ channels
Sulphaphenazole	CYP epoxygenase	Highly specific of CYP 2C9
Fluconazole	CYP epoxygenase	Can inhibit other CYP isoforms at >10^−4^ m
MSPPOH	EETs synthesis inhibitor	Highly specific
14,15-EEZE	EETs antagonist	Inhibits the vasodilator action of all EETs regioisomers

K^+^: potassium, SK_Ca_^+^: small calcium-dependent potassium channels, IK_Ca_^+^: intermediate calcium-dependent potassium channels, BK_Ca_^+^: large calcium-dependent potassium channels, Kv: voltage dependent potassium channels, K_IR_^+^: inwardly rectifying potassium channels, BaCl_2_ barium chloride, KCL: potassium chloride, CYP: cytochrome, 20-HETE: 20-hydroxyeicosatetraenoic acids, MSPPOH: N-(methylsulfonyl)-2-(2-propynyloxy)-benzenehexanamide, EETs: epoxyeicosatrienoic acids, and 14,15-EEZE: 14,15-Epoxyeicosa-5(Z)-enoic Acid.

**Table 2:** Human vascular territories with characterized EDHF activity.

Vascular territory	EDHF	Pharmacological agents used
*Preclinical studies*		
Coronary arterioles [8, 9, 29, 73, 118]	H_2_O_2_, K_Ca_^+^ channels, CYP450 metabolites	Catalase, KCl, charybdotoxin + Apamin, polyethylene glycol catalase, KCl, charybdotoxin, 7-octadecynoic acid
Internal Mammary artery [6, 62]	11,12-EET	17-octadecynoic acid, N-methylsulfonyl-6-(2-propargyloxyphenyl)hexanamide4,15-epoxyeicosa-5(Z)-enoic acid
Gastroepiploic arteries [4]	K_Ca_^+^ channels	KCl
Mesenteric artery [66, 119]	H_2_O_2_, Gap junctions, superoxide dismutase, H_2_O_2_	Catalase, 18 alpha-glycyrrhetinic acid, Tiron (cell-permeable SOD-mimetic), catalase
Renal artery [10]	K^+^, K_Ca_^+^ channels	KCl, charybdotoxin, and apamin
Subcutaneous resistance arteries [7]	CYP450 metabolites, K_Ca_^+^ channels	Ketoconazole
Subcutaneous resistance arteries [120] (subcutaneous fat biopsies of healthy pregnant women)	Connexin 43 Gap junctions.	Connexin mimetic peptides
Visceral fat arterioles	H_2_O_2_	Polyethylene glycol catalase
Umbilical vein endothelial cells [85, 121]	SK_Ca_^+^ channels, IK_Ca_^+^channels, H_2_O_2_	Apamin and charybdotoxin/triarylmethane-34
Thyroid arteries [122]	K_Ca_^+^ channels, K_IR_^+^ channels, Na^+^/K^+^ ATPase	Iberiotoxin, charybdotoxin, apamin glibenclamide, and barium

*Clinical studies*		
Forearm microvasculature [63]	CYP450 metabolites	KCL, miconazole
Forearm microvasculature [30, 32, 123]	K_Ca_^+^ channels	TEA
Forearm microvasculature [124]	C-type natriuretic peptide	C-type natriuretic peptide, TEA
Forearm microvasculature [104] (hypertensive patients)	CYP450 2C9	Sulfaphenazole
Forearm conductance vessel [31, 125, 126]	CYP 2C9 metabolites, K_Ca_^+^ channels	Sulfaphenazole, TEA, fluconazole
Thigh skeletal muscle vessels [127]	CYP450 2C9	Sulfaphenazole

H_2_O_2_: Hydrogen Peroxide, K^+^: potassium, SK_Ca_^+^: small calcium-dependent potassium channels, IK_Ca_^+^: intermediate calcium-dependent potassium channels, K_IR_^+^: inwardly rectifying potassium channels, KCL: potassium chloride, CYP: cytochrome, and EETs: epoxyeicosatrienoic acids.
